# Empowering women for health security in India: a One Health cascade training model for strengthening biosecurity through women veterinarians and farmers

**DOI:** 10.3389/fpubh.2026.1903744

**Published:** 2026-08-26

**Authors:** Ravina Tadvi, Jessica Taaffe, Ashley Tseng, Shrankhala Mishra, Pratiksha Pundir, Shailee Patil, Sandul Yasobant, Deepak Saxena, Falgunee K. Parekh

**Affiliations:** 1 Centre for One Health Education, Research & Development (COHERD), Indian Institute of Public Health Gandhinagar (IIPHG), Gandhinagar, Gujarat, India; 2 EpiPointe, Cary, NC, United States; 3 Department of Public Health Science, Indian Institute of Public Health Gandhinagar (IIPHG), Gandhinagar, Gujarat, India; 4 Global Health, Institute for Hygiene & Public Health, University Hospital Bonn, Bonn, Germany

**Keywords:** biosecurity, India, One Health, women, zoonotic disease

## Abstract

**Introduction:**

Zoonotic diseases remain a significant public health threat in India, where human–animal interactions are frequent due to its dense population and distinct agricultural economy that lends itself to an increased risk of zoonotic spillover of high-consequence pathogens. Despite policy initiatives promoting One Health approaches, barriers like limited awareness and inadequate biosecurity practices persist. Women farmers play crucial yet under-recognized roles in livestock management but have limited access to training on best biosecurity practices due to limited engagement with male veterinary health professionals, who primarily conduct field visits in India. Women veterinary health professionals (WVHPs) can operationally engage better with WFs but similarly face barriers to entry for fieldwork positions and have limited access to training for professional growth.

**Methods:**

To address this gap, we designed and implemented an innovative cascade-training model with the goal of strengthening detection and prevention of high-consequence pathogens by linking WVHPs and WFs. We employed a cascade-training model beginning with training of WVHPs on biosafety, biosecurity, One Health, and community engagement and equipping them with the tools and skills to train WFs. These trained WVHPs implemented local training sessions for WFs using interactive tools like case studies and games. Acquired knowledge metrics were assessed through pre- and post-training assessments and focus group discussions (FGDs).

**Results:**

We trained 38 WVHPs, and knowledge scores improved from 10.6 ± 2.49 to 11.3 ± 2.47 when given the same questions in pre- and post-assessments. The FGDs demonstrated WVHPs’ understanding of WF knowledge and issues faced. Three overarching themes emerged regarding WF capacity building: knowledge gaps and high-risk practices, structural and sociocultural barriers, and pathways for effective community engagement/knowledge transfer. Of the 152 WFs trained, 79.9% could identify clinical signs of animal sickness, and 85.9% recognized integrated prevention practices. However, gaps in zoonotic disease awareness and sanitation practices were evident.

**Conclusion:**

The cascade-training model demonstrated that engaging women professionals as both practitioners and trainers can advance community-level biosecurity practices and disease detection. Embedding such women-centred approaches within national One Health strategies can strengthen India’s biosecurity systems and contribute to global health security goals.

## Introduction

Emerging and re-emerging zoonotic diseases pose major threats to global health security, particularly in countries where human–animal interactions are highly prevalent. India, with its dense population, vast livestock sector, and porous interfaces between animals, humans, and the environment, combined with rapid urbanization, is especially vulnerable to zoonotic spillover events and outbreaks caused by high consequence pathogens (HCPs) (1). Zoonotic pathogen prevalence in Indian livestock varies substantially by pathogen type, species, and region, ranging from 2 to 43% depending on the disease. The prevalence rates of 6–35% for bacterial pathogens (*Brucella*, *Clostridium*, *Escherichia coli*, *Listeria*, *Salmonella*, *Staphylococcus*, and *Streptococcus suis*) across India over 2010–2023 (2). In the North Eastern Region, Barman et al. (3) reported brucellosis prevalence of 2–18% across different livestock species, with classical swine fever at 31% and porcine circovirus at 43%. India has initiated the prioritization of zoonotic diseases at the national level using participatory methods like the One Health Zoonotic Disease Prioritization Tool (4). It has also rolled out the Roadmap to Combat Zoonoses in India (RCZI) initiative, which has systematically identified research priorities for zoonoses control and emphasizes the need for multisectoral collaboration and “actionable research” (5). Key zoonoses prioritized in India include rabies, brucellosis, avian influenza and Crimean-Congo hemorrhagic fever (4). Despite these policy efforts and recognition of the importance of One Health approaches, there are challenges in translating effective cross-sectoral action, particularly at the local level. Strengthening community-level capacity in biosecurity, One Health, detection, and prevention practices to mitigate risk of zoonotic spillover is especially fundamental for India where the majority of the human population lives in rural areas and has daily contact with both livestock and wildlife (1). International frameworks such as the Global Health Security Agenda (GHSA) and The Quadripartite One Health Joint Plan of Action emphasize the integration of community engagement and cross-sectoral coordination. However, practical, evidence-based models demonstrating how these can be operationalized at the community level remain scarce.

Women constitute a substantial proportion of India’s agricultural workforce and play a pivotal role in daily animal husbandry. Women farmers (WFs) are typically responsible for animal feeding, cleaning, and care, bringing them into close daily contact with livestock and potential zoonotic pathogens. As such, WFs function as frontline gatekeepers at the animal–human interface and are critical to improving the detection and prevention of zoonotic pathogens in India. Despite this central role WFs face significant challenges including limited access to training, resources, and decision-making opportunities (6).

There are also socio-cultural barriers that may prevent the empowerment of WFs in a biosecurity role. In rural communities India, traditional gender roles may limit interactions between women and male professionals. As field visits are mostly conducted by male veterinary professionals, who also greatly outnumber females in the profession, this socio-cultural norm could limit WFs’ direct engagement with veterinary practitioners and reduce their access to training, information, and resources.

Due to these sociocultural gender norms in India, women veterinary health professionals (WVHPs) may be able to engage better with WFs. Also, they often have positions in clinical or laboratory settings, in which biosecurity practices, detection, and prevention are key aspects of their job responsibilities. WVHPs, with their knowledge base combined with their ability to better engage with WFs, are distinctly positioned to be key integrators of enhancing capacity in One Health, biosecurity, and detection of zoonotic/emerging pathogens at the community level. However, WVHPs similarly face barriers-to-entry for field work positions and have limited access to training for professional growth. Thelwall et al. (7), noted that veterinary science in India has much lower female representation compared to the USA, suggesting systemic gender imbalance in the field. This gender imbalance is further reflected in limitations for career progression available to WVHPs. While veterinary health training does exist, WVHPs are often not selected by the leadership of their institutions to participate in continuing education training opportunities.

To address this knowledge gap, we designed and implemented an innovative cascade-training model linking WVHPs and WFs to strengthen the detection and prevention of HCPs. The model positioned WVHPs as integrators of biosecurity and One Health practices who mentor WFs at the community level, while empowering WFs to improve daily biosecurity behaviors and serve as champions of infection prevention within their communities. Through this approach, we aimed to establish a sustainable network of women professionals that facilitates collaboration and information sharing, thereby advancing a functional One Health approach to identifying and mitigating HCP outbreaks in India. This paper describes the design, implementation, and evaluation of the cascade-training model and presents key findings from quantitative and qualitative assessments, lessons learned, and implications for scaling within national and global biosecurity frameworks.

## Methods

### Study design and approach

**Figure 1:**
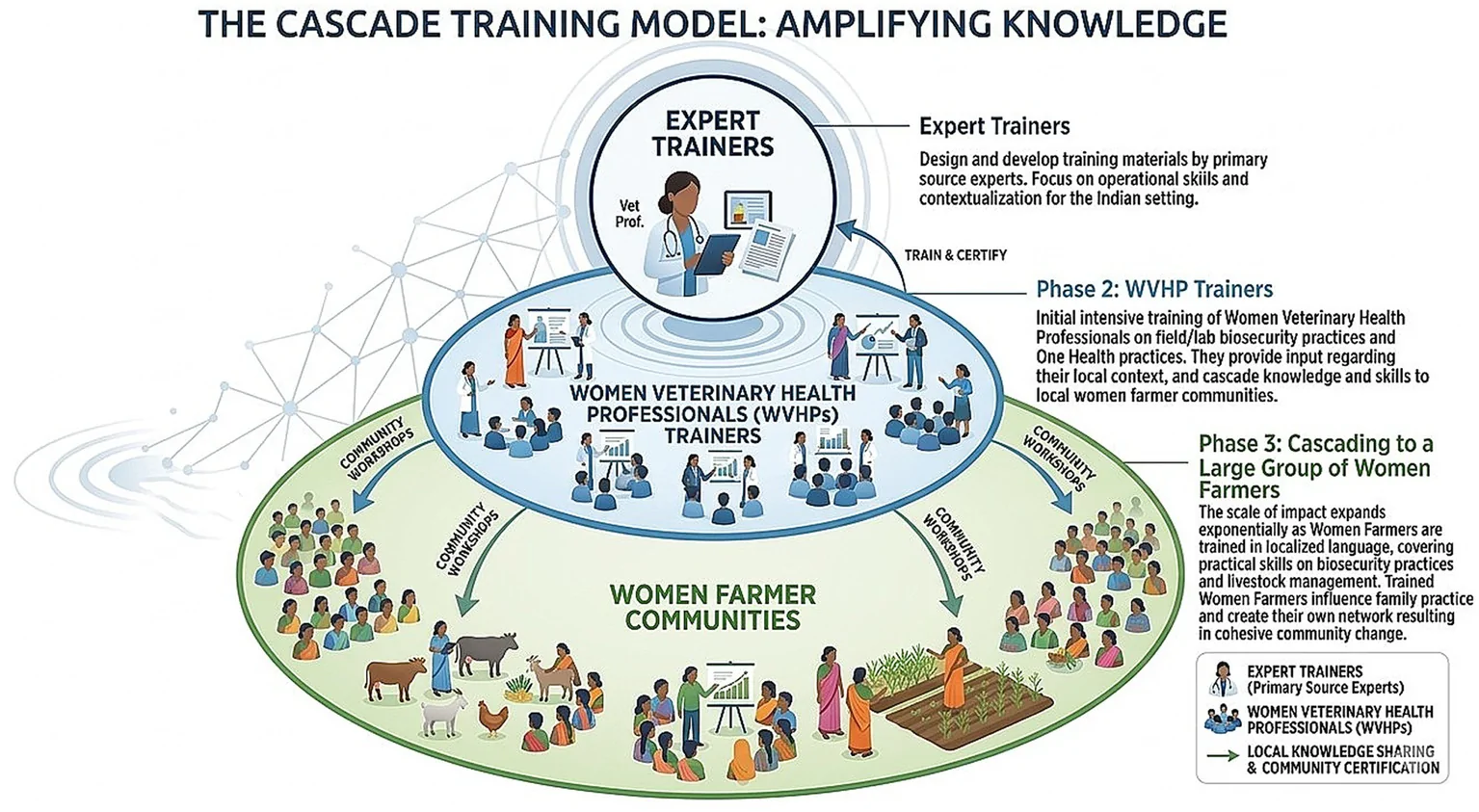
Two-tiered capacity building training model. This figure demonstrates the three-tiered cascade training model aimed at developing WVHPs into trainers for dissemination of knowledge to WFs. This figure was developed with the assistance of generative AI (Gemini).

The model was designed as a two-tiered capacity building cascade training model using a participatory method in which WVHPs are first trained as master trainers (integrators) who then turn around and train WFs within their respective communities (Figure 1). In India, veterinary professionals, particularly those employed by the State Department of Animal Husbandry and Fisheries, are assigned to work in specific sub-districts and villages. Strengthening engagement between WVHPs and WFs, and building a network across them, can therefore enhance capacity at the animal–human interface in the highly localized communities where early detection and mitigation of HCP outbreaks are most critical.

The cascade-training model included three types of workshops; two targeted WVHPs and one targeted WFs. All training materials were specifically developed for this effort and made available online for participants to revisit or review any of the topic areas. The first type of workshop, WVHP Professional Training (WVHP PT), aimed to enhance the knowledge of WVHPs in biosecurity and One Health practices as applicable in laboratory, clinic, and field settings utilizing a case-based approach, thereby preparing them as future integrators of best practices. WVHPs were trained in the following topics during a two-day in-person workshop: Introduction to Biological Risk Assessments, Facility and Laboratory BS&S Plans, Field Biosecurity and Operations, One Health Epidemiology, PPE Use, Sample Collection and Transport Procedures, and Disease Reporting. Training sessions on these topic areas were integrated with a progressive case study designed to simulate a real-life scenario, moving stepwise from detection to sample collection and ultimately disease reporting. For example, participants would receive training on One Health, field operations, and sample collection and transport, and then participate in a case study that highlighted a WVHP encountering a possible HCP, and how they used appropriate biosecurity and biosafety measures to assess the case and collect the sample. Then, WVHPs would receive training in laboratory BS&S, and the case study would continue to see how sample is handled in a laboratory setting, etc. During this first workshop, we also conducted focus group discussions (FGDs) with WVHPs to gain insights on barriers and challenges they face in engaging with the community, especially WFs. Information discussed during these FGDs was utilized to inform development of training workshop for WFs.

The curriculum of the second type of workshop, WVHP Community Engagement Training, (WVHP CE), focused on preparing WVHPs to train WFs in field biosecurity practices, and establish and maintain engagement with them. During this one-day in-person workshop we trained WVHPs on community engagement, and delivery and use of the training materials and content designed for WF training workshops. At the end of these two workshops, WVHPs were prepared to serve as trainers and implement training workshops for WFs within the communities that they service. All training materials for WVHPs were developed in English.

The third type of workshop was specifically designed for WFs. We developed accessible training materials in the local language, and to meet the training needs of WFs with varying literacy levels. Curriculum for WFs focused on the following topics: biosecurity and biosafety practices to use in their daily animal husbandry responsibilities, best practices to prevent infection, identifying possible illness in their animals, when to contact a veterinary professional, and disease reporting. Materials were developed in a variety of formats including posters, games, short videos, and guided questions for focus group discussions.

### Study setting

This cascade-training model was piloted in the state of Gujarat, India, because of three main reasons: First, the state has a significant livestock population of 26 million animals and a well-developed network of veterinary institutions (8, 9). Second, a critical study revealed that 87.7% of veterinarians urgently need occupational hazards and safety training (10). Lastly, the state accounts for 5.36% of India’s livestock population, contributes 7.08% to India’s total milk production, and accounts for 1.22% of the country’s meat production. The two-day workshops for WVHPs were conducted at university institutions—one at India Institute of Public Health, Gandhinagar (IIPHG) and one at College of Veterinary Science and Animal Husbandry, Anand.

### Participants

A total of forty-one WVHPs were selected to participate in the training program with the assistance of the local public veterinary university, Kamdhenu University Gandhinagar. As they have five affiliated veterinary colleges throughout the state, we were able to select WVHPs from different areas representing the diverse regions of Gujarat. WVHPs with varying levels of experience were also nominated to participate in the training, but all participants had a similar educational background and completed bachelor courses in veterinary science.

During the two-day workshop, WVHPs were tasked with identifying WFs within the respective communities in which they work, which also served to begin the process of building community level WVHP-WF networks. With the assistance of WVHPs, a total of 152 WFs were identified and trained during 5 workshops.

### Assessment process

We used both quantitative and qualitative assessments to evaluate the impact of this novel cascade-training model. IRB approval was obtained to administer the assessments to WVHPs and WFs.

#### Data collection and analysis of WVHPs

Pre-and post-training assessments for the WVHPs were conducted using a structured questionnaire to evaluate knowledge related to pathogen detection, biosecurity, zoonotic diseases, laboratory safety, and One Health principles. Assessments were administered via an online learning management system that allowed for automatic grading. Questionnaire items covered diagnostic methods, disease reservoirs, biological hazards, distinctions between lab safety and field biosecurity, and occupational hazards faced by veterinarians, as well as core biosecurity concepts including risk assessment, personal protective equipment (PPE), and facility design. The pre-assessment comprised of 16 questions, while the post-assessment included the same set of 16 questions plus14 additional items, for a total of 30 questions. Responses were scored dichotomously (correct = 1, incorrect = 0), and descriptive analyses were conducted using Jamovi version 2.5.3.

FGDs were conducted with 36 WVHPs to obtain their insights in WF knowledge and practices in biosecurity and animal management, and identify effective training methods for WFs that would be feasible in their districts. A total of 36 WVHPs were subdivided into four distinct groups, ensuring 9 participants in each group. The key themes were challenges and benefits associated with different community engagement methods, farm biosafety improvements, and identifying local issues and barriers to engagement of WFs, such as societal or logistical challenges. The discussion was conducted verbatim, and audios were recorded for analysis. All recordings were transcribed and coded using inductive logic in MAXQDA 24. The thematic coding facilitated the identification of key patterns and insights, which were subsequently synthesized into the results. The informed consent was collected from all participants before conducting the assessments and FGDs.

#### Data collection and analysis of WFs

Similar to WVHPs, pre- and post-training assessments were also designed for WFs to measure knowledge on biosecurity and animal health and management practices. During the workshops, we found that many WFs had difficulty completing the pre-assessment for several reasons, including literacy issues and lack of familiarity with multiple choice tests. As a result, the trainers verbally administered the pre-assessments to the participants and recorded their responses, and the post-assessment was not conducted due to feasibility and time limitations.

The assessment aimed to obtain a detailed understanding of practices and perceptions related to animal health and zoonotic disease prevention. Ten multiple-choice questions covered key areas such as identifying risk factors for animal sickness, cleaning, and hygiene practices for sheds and milking utensils, recognizing signs of animal illness, and appropriate responses to sickness, including isolating affected animals and consulting a veterinarian. Additional questions assessed awareness of zoonotic diseases, unsafe practices that could lead to human illness, and the role of vectors like flies, ticks, and mosquitoes in disease transmission. The assessment also explored best practices for infection prevention, such as providing high-quality feed, ensuring regular deworming and vaccination, and seeking veterinary advice.

The assessment was translated to and administered in the local language, Gujarati. Verbal consent was obtained from all participants before data collection.

## Results

We identified 42 WVHP participants representing different regions of Gujarat to participate in the training program in June 2024. The 41 participants were divided into two cohorts for the two-day WVHP PT workshop to maintain manageable group sizes and ensure regional accessibility across regions of Gujarat. The workshop for cohort 1 was conducted at IIPHG and the workshop for cohort 2 was conducted at College of Veterinary Science & Animal Husbandry, Anand.

One-day WVHP CE workshops were conducted in July–August 2024, and were held at four locations in proximity to participants’ work areas to maximize accessibility. The locations of these WVHP CE workshops were in the following cities/towns: Dantiwada, Himmatnagar, Navsari, and Junagadh. In September 2024, one-day training sessions for WFs by WVHPs were conducted at similar locations.

### Result of WVHPs: pre and post test

Table 1 presents the results of a pre-test and two post-tests conducted on 38 WVHPs participants, where a correct and incorrect answer received scores of 1 and 0, respectively. The mean and standard deviation (SD) for the pre-test (total of 16 questions) was 10.6 ± 2.49, which increased to 11.3 ± 2.47 in the post-test with the same 16 questions, indicating a performance improvement. However, the post-test with 31 questions, scored with partial credits (0.5), showed a mean of 10.6 ± 2.43, similar to the pre-test.

**Table 1:** Univariate analysis of pre and post test responses captured during the training of women veterinary health professionals in Gujarat during 2024.

Statistical measures	Pre test (16) score 1	Post test (16) score 1	Post test (31) score 0.5	14 additional questions
*N*	38	38	38	38
Mean ± SD	10.6 ± 2.49	11.3 ± 2.47	10.6 ± 2.43	9.79 ± 2.95
Median	10 (11.8–9)	11.5 (13–10)	10.5 (12–9.5)	9.5 (12–8)
25th percentile	9	10	9.5	8
50th percentile	10	11.5	10.5	9.5
75th percentile	11.8	13	12	12

The additional 14 questions were also assessed separately; the mean score was 9.79 ± 2.95. The median scores followed a similar trend, increasing from 10 (pre-test) to 11.5 (post-test for 16 questions) before declining to 10.5 for the 14-additional questions test and 9.5 for the subset. These results suggest that participants performed better when retested on the same set of 16 questions but found the additional or subset of questions relatively challenging.

### Result of WF pre-test

While the originally planned pre-and post-assessments were not conducted due to literacy and test-taking issues among WFs, trainers were able to verbally administer the pre-assessment to WFs, enabling us to gain an understanding into current WF knowledge and practices related to animal health and management. Supplementary Table 1 presents the results of this assessment.

Most participants (79.9%) correctly answered that animal sickness can be identified through signs such as lack of eating, abnormal behavior, fever, and diarrhea. When asked what action is taken when animals are sick, 60% reported that they should both consult a veterinarian and isolate the sick animals. Telephonic communication was the most preferred method 51.1% for contacting veterinarians. More than half of the respondents (55.2%) reported that gender of the veterinarian does not matter for animal treatment, but 22.8% reported preference of a male veterinarian (Supplementary Table 1).

Hygiene practices and prevention strategies were widely recognized as critical for animal health. While only 36.5% of women correctly responded that introducing new animals could cause illness in their animals, most respondents (85.9%) reported that quality feed, deworming, vaccination, and veterinary advice, are all essential for preventing and controlling infections Knowledge of best cleaning practices were largely varied, with 57.2% using soap powder and 11.7% relying only on water, while fewer 29.7% respondents used soap powder containing disinfectants. Regarding knowledge of best post-milking practices, most WFs (79%) suggested waiting 10–30 min before allowing animals to sit, reflecting awareness of maintaining hygiene.

The assessment also revealed gaps in awareness about zoonotic diseases. Nearly half (49.3%) incorrectly responded that animals cannot make humans sick, despite 28.6% of participants acknowledging the bidirectional transmission of diseases. In contrast, most respondents (57.7%) recognized that diseases could be transmitted via vectors like flies, mosquitoes, and ticks or through inhalation of contaminated air. Unsafe practices such as drinking raw milk, eating undercooked meat, and not washing hands after handling animals were correctly identified as risks for illness by the majority of participants (69.3%). These findings underscore the need for targeted educational programs for WFs to address misconceptions and promote best practices in animal health and zoonotic disease prevention.

### Results of WVHPs: focus group discussion

The FGDs revealed that WVHPs have a strong understanding of farmers’ knowledge, practices, attitudes and challenges in animal husbandry and health care. Three overarching themes from the discussion were identified. Specific comments that highlight these themes are also shown. These insights can be utilized to improve the training materials for WFs in the future.

#### Knowledge gaps and high-risk practices at the community level

When the WVHPs were asked about the behavior and attitudes of farmers residing in their area towards biosecurity, the majority of them responded that it depends on farmers’ level of education. Farmers in Gujarat demonstrated varied levels of understanding based on literacy. Literate farmers actively engaged in diagnostic and treatment processes, often guiding veterinary procedures and making informed decisions about their animals’ healthcare.

*“Farmers in Gujarat are intelligent. They directly ask us to do ELISA or RBPT when diagnosing diseases. However, they do not know the correct method of sample collection. They often collect it in normal bottles and bring contaminated samples for testing.”*

However, gaps in knowledge remain prevalent among less literate farmers, who tend to rely more on traditional methods or myths, i.e., vaccination cause abortion and reduce milk production rather than evidence-based practices. WVHPs noted a significant lack of awareness about zoonotic diseases and biosecurity practices during their field visits. When asked to share their views on which areas need improvement at the community level, they suggested common issues such as improper waste disposal, handling of animal waste, and myths about vaccination. Demonstrating the benefits of hygiene and vaccination through practical examples and success stories was suggested as an effective strategy.

*“Farmers handle brucellosis material with bare hands, which increase chance of producing more positive cases of brucellosis.”*

*“They believe vaccination causes abortion, so they refuse it outright.”*

#### Structural and sociocultural barriers shaping WF engagement

WFs face substantial challenges in attending any training due to their disproportionate household responsibilities and the prevailing social stigma against their involvement.

*“Women are too busy with chores to attend sessions, and when they do, men often speak on their behalf.”*

Practical challenges, such as lack of time, seasonal constraints, and limited access to technology, significantly hinder WF participation in training opportunities. In addition to the demands of their agricultural work and household responsibilities, the seasonal nature of farming activities made it challenging for some to engage throughout the year consistently.

*“If training overlaps with milking or harvest times, no one will attend.”*

Additionally, caste divisions within the community further limited WF group cohesion and collective participation.

*“Villages are often divided by caste, so we must approach communities carefully through their local leaders.”*

Furthermore, digital engagement methods, such as WhatsApp groups, also encountered barriers. This limited access to digital tools and platforms posed a significant obstacle for many women, who were a key target audience for the program. The program organizers recognized the need to explore a combination of approaches to address these challenges and improve engagement. Scheduling training sessions at optimal times, when WFs are more available, and supplementing digital methods with more traditional outreach techniques, such as in-person meetings and workshops, could help overcome the practical barriers and ensure broader participation.

*“Most women do not have smartphones; only their husbands do, which causes miscommunication.”*

Finally, WVHPs also faced initial skepticism from farmers, who often preferred to work with male professionals. This was due to prevailing gender norms and societal expectations favoring male authority figures in veterinary medicine. However, as the female veterinarians consistently engaged with the farming community and demonstrated their competence through tangible results, the farmers’ trust and acceptance of WVHPs grew.

*“Initially, they doubted us, but when they saw results from our treatments, they started trusting us.”*

#### Pathways for effective community engagement and knowledge transfer

WVHPs discussed possible strategies to enhance WF participation in training opportunities. Providing incentives and involving trusted community leaders like sarpanch (village heads) and livestock inspectors could be effective in encouraging broader participation. Engaging these local leaders to help build trust within the community and ensured broader outreach for the training activities.

*“They ask, ‘What will we get at the camp?’ They’re more interested in kits or tools than just information.”*

*“The sarpanch can announce training through megaphones, and livestock inspectors can guide farmers in vernacular language.”*

The use of simple, culturally relevant materials, including local language booklets, pictorial aids, and videos, make training more accessible. Interactive methods such as role plays and demonstrations.

*“If the materials are in Gujarati with more pictures than text, both literate and illiterate farmers can understand.”*

*“Presenting a story where a farmer’s practices led to healthier animals motivates others to follow suit.”*

WVHPs noted that there is strong interest among WFs in a range of practical topics, including poultry farming, vaccination schedules, and proper hygiene practices for their livestock. They also highlighted the critical need for training on biosecurity measures, such as proper waste disposal techniques and strategies to control antimicrobial resistance. These areas were seen as essential for improving disease prevention and animal health outcomes within the communities.

*“If humans get sick, we spend up to 1 lakh, but a new animal costs just 10,000. It’s cheaper to replace them.”*

Success stories and practical demonstrations resonated strongly with farmers. Case studies highlighting the economic benefits of better practices, such as proper vaccination and hygiene, were seen as effective tools for knowledge transfer.

*“Farmers trust us more when we show results—like when following advice improves milk production.”*

### Key observations and issues for WVHP PT workshop

The trainers for the WVHP PT workshop noted several key observations and issues that can help to improve the training program in the future. Notable observations were high participation and interactivity and interest in case study discussions. WVHP participants were highly interactive during the sessions and were actively engaged with the training content, activities, and discussion. They especially showed keen interest in delving deeper into case study discussions and provided suggestions on aspects of case studies that could be shared with farmers to enhance farmer knowledge.

Some key issues of the WVHP PT workshop included time management and applicability of session content. Case study discussions took more time than expected as participants were highly engaged with these, which presented an issue in timely completion of all sessions. Sessions on PPE usage, sample collection, and transport procedures may be less necessary as participants already had considerable knowledge on these topics. In contrast, additional sessions to delve more in depth on topics of disease reporting and outbreak response may be more necessary as WVHP participants had many questions on these topics.

Observations from the subsequent WF training workshops further validated several issues identified during the WVHP PT workshop. In particular, WVHPs’ early concerns regarding literacy constraints, sociocultural barriers, and time limitations among WFs were consistently observed across all four WF training sites. Many WFs reported that they had never previously attended animal health or biosecurity training due to lack of invitation, caste-based exclusion, or competing household and agricultural responsibilities, underscoring the structural nature of these barriers rather than a lack of interest.

WVHPs’ emphasis on the need for low-literacy, participatory training approaches was reinforced during WF workshops, where interactive methods such as games, case discussions, and demonstrations generated high engagement, while written assessment tools proved challenging for many participants. These observations suggest that future WVHP PT workshops should further strengthen skills in adaptive community engagement, alternative assessment methods, and facilitation techniques tailored to varying literacy levels.

Additionally, WVHPs’ initial observations regarding limited awareness of zoonotic disease transmission among WFs were corroborated during WF trainings, particularly around risks associated with raw milk consumption, vaccination hesitancy, and unsafe handling of animal waste. These findings highlight the importance of equipping WVHPs not only with technical biosecurity knowledge but also with practical communication strategies to translate One Health concepts into locally meaningful messages.

### WVHP participant feedback

WVHPs also provided feedback regarding the workshop and training program. Most notably was that many of the participants expressed that this was their inaugural experience participating in such a workshop and their first time attending professional training since completing their degree. While professional training is available to veterinary professionals in India through supervisor nomination or selection, this feedback demonstrates that these opportunities are rarely offered to WVHPs. WVHPs stated they enjoyed the interactive nature of the sessions that allowed for two-way conversation, which enhanced the overall learning experience. They especially enjoyed the inclusion of case studies which added depth to the topic area being addressed. WVHPs also expressed that the training was helpful in gaining insights into the new direction of One Health, underscoring the significance of the discussions and interactions with fellow WVHPs and the speakers. Finally, WVHPs appreciated the emphasis on biosafety and biosecurity measures and acknowledged the importance of these measures in their professional practice.

Feedback from WVHP participants following implementation of WF training workshops indicated that the cascade-training model enhanced their confidence and perceived professional role within their communities. Several WVHPs noted that leading WF workshops provided them with opportunities to apply skills gained during the PT and CE trainings, particularly in community engagement, facilitation, and adapting technical content for non-technical audiences.

WVHPs reported that sustained interaction with WFs helped build trust and credibility, even in contexts where female veterinarians initially faced skepticism. Observing WFs’ active participation and willingness to ask questions during training sessions reinforced WVHPs’ perception of women farmers as frontline actors at the animal–human interface. WVHPs also reflected that their involvement helped bridge gaps in awareness about available veterinary services, as many WFs were previously unaware of nearby animal healthcare facilities.

Importantly, WVHPs highlighted that participation in this program represented a rare opportunity for professional development and leadership, noting that similar training opportunities are infrequently available to women veterinarians. The experience of serving as trainers and mentors strengthened their sense of professional agency and reinforced the value of women-centered approaches for advancing biosecurity and One Health implementation at the community level.

## Discussion

This study aimed to design and pilot an innovative cascade training model to enhance biosecurity and biosafety knowledge and practices in India by targeting and connecting two important groups at the human-animal interface: women veterinarians and women farmers. Other studies have demonstrated that veterinarians are recognized as key informants for farmers regarding biosecurity measures, yet their ability to effectively disseminate knowledge and promote compliance relies heavily on adequate training (11, 12). In addition, studies have shown that training farmers can result in improvements in disease morbidity and mortality. A study of 100 Punjab dairy farmers found that those attending dairy training had significantly lower clinical mastitis incidence (33.33% affected vs. 40% in untrained farmers, *p* < 0.05) (13). In poultry farms across Telangana, trained farmers (*n* = 30) demonstrated superior disease management practices, with lower mortality (4.8% vs. 9.6%) and morbidity (11.2% vs. 21.5%) compared to untrained farms (14). Training on animal disease management was significantly associated with improved knowledge about brucellosis transmission in Indian dairy farmers (15).

Our cascade training model is among the first to systematically operationalize biosecurity and One Health capacity building by establishing women professionals as technical experts in India, community trainers, and change agents within their communities. Moreover, the cascade training model enables the development of functional networks between and among WVHPs and WFs which will contribute to the long-term sustainability of improved biosecurity practices.

The pre-and post-training assessment findings reveal a modest, but significant, improvement in veterinarians’ knowledge of biosecurity and biosafety practices (score increase from 10.6 ± 2.49 in the pre-test to 11.3 ± 2.47 in the post-test). The observed improvement underscores the potential of targeted, case-study based workshops to enhance understanding and awareness among WVHPs and its applicability to clinical, laboratory, and field settings. However, a more comprehensive post-test incorporating 31 questions did not demonstrate participants’ ability to apply concepts to different types of biosecurity related questions and scenarios. These findings indicate the need for integrating additional training topics to this program and a strategy for sustainability, such as a series of workshops rather than just one short, intensive workshop.

The WF assessment revealed a clear dichotomy between animal health awareness and biosecurity knowledge in their daily animal management practices. A majority of WFs demonstrated strong recognition of livestock illness, with 79.9% identifying sickness based on common clinical signs and 60% reporting consultation with veterinarians and isolation of sick animals to reduce disease spread. In contrast, fewer than one-third (29.7%) reported using disinfectants for hygiene and nearly half, 49.3% lacked awareness of zoonotic diseases, representing a significant public health concern. These findings are consistent with previous studies emphasizing the health risks posed by close interactions with livestock, particularly among women farmers who often have limited access to education on biosecurity and hygiene (16).

This study underscores the importance of biosecurity training for both WVHPs and WFs in preventing and mitigating zoonotic disease outbreaks. Effective communication between veterinarians and farmers is essential as miscommunication often leads to poor biosecurity practices (17). Veterinarians’ competence and communication skills significantly influence farmers’ adoption of biosecurity measures (18). As veterinarians gain experience, their comfort and capability in delivering biosecurity advice improve, resulting in better farmer engagement. Future efforts should focus on developing strategies for effective communication and knowledges transfer for local populations improving veterinary education on biosecurity, and strengthening the veterinarian-farmer relationship (17, 18). This is especially critical for the WVHP-WF relationship as WFs are often the first responders to livestock illness given their routine caregiving roles and close daily contact with animals.

The FGDs demonstrated that WVHPs have a strong understanding of the knowledge, attitudes, practices, and challenges of farmers and are aware of specific issues and barriers facing WFs in their respective locales. Moreover, it also allowed us to understand WVHPs’ level of knowledge regarding the challenges WFs may face in implementing field biosecurity practices. The key findings from FGD emphasize the importance of tailored training programs, particularly for women farmers who cannot attend training sessions due to various social barriers, i.e., disproportionated household responsibility. Other researchers also highlight similar difficulties for women, such as time constraints due to household responsibilities and male dominance in decision-making (6, 19). Huynh et al. (20) also studied that women often get limited exposure to social activities, which hinders their participation.

The main aim of conducting a FGD was to understand the local issues women farmers face in animal husbandry, their level of participation in the training, and how this innovative approach can enhance their accessibility to training programs. Additionally, the group discussions with WVHPs facilitated the arrangement of local training sessions for women farmers, including presentations in Gujarati dialect verbatim language, case study video discussion, and snake and ladder game. To sum up, most of the studies did not encounter solutions for such constraints faced by the women (19–21); however, we addressed this issue and reported exceptional participation by women farmers in this study.

This study has several limitations. The small sample size (38 WVHPs & 152 WFs) and its focus on a single geographic region (Gujarat, India) may limit the generalizability of the findings. The limited scope of the pre-and post-assessments and inability to conduct long-term follow-up may not fully capture enduring knowledge retention or the complexity of implementation of learned biosecurity practices. Additionally, variations in training quality across different regions or trainers were not assessed. Future research should address these limitations with larger, more diverse training populations, more extended follow-up periods, and more comprehensive evaluation tools.

## Conclusion

This study demonstrates the potential of a targeted training model that engages women professionals at multiple levels of the animal–human interface for a strategic and sustainable approach to advancing biosecurity and health security in India. By empowering WVHPs to train and mentor WFs, the cascade model can successfully translate biosecurity practices and One Health principles to actionable community practice.

## Data Availability

The original contributions presented in the study are included in the article/Supplementary material, further inquiries can be directed to the corresponding author.
