# Supplementary material for: Empowering women for health security in India: a One Health cascade training model for strengthening biosecurity through women veterinarians and farmers
Source: Front Public Health. 2026 Aug 26;14:1903744. doi: 10.3389/fpubh.2026.1903744 (PMC13556655; doi:10.3389/fpubh.2026.1903744)
Supplement: Supplementary file 1 [file Table_1.DOCX]

Supplementary Table 1: Univariate analysis of the responses captured from the Women Farmers trained under this program in Gujarat during

| **Question** | **Category** | **n=152 (%)** |
| --- | --- | --- |
| How would you tell if your animal(s) is/are sick? | a) Animal has stopped eating/drinking | 24 (17.3) |
|  | b) Animal shows abnormal behaviour | 1 (0.7) |
|  | c) Signs like fever, diarrhoea | 3 (2.2) |
|  | d) All of the above | 111 (79.9) |
| If your animal is sick and shows abnormal signs/behavior, what should you do? | a) Wait to see if the animal gets better | 9 (6.4) |
|  | b) Report to the veterinarian immediately | 43 (30.7) |
|  | c) Separate the sick animal from healthy animals | 4 (2.9) |
|  | d) b and c | 84 (60) |
| **In case you have to contact a veterinarian, how would you do that?** | a) I would call him/her telephonically | 72 (51.1) |
|  | b) I may visit the village animal dispensary | 25 (17.7) |
|  | c) I ask my family member/neighbour to call him | 35 (24.8) |
|  | d) Any other: | 9 (6.4) |
| In case you have an option to contact a male or female veterinarian, who would you be more comfortable to contact? | a) Female veterinarian than a male veterinarian | 31 (21.4) |
|  | b) Male veterinarian than a female Veterinarian | 33 (22.8) |
|  | c) It does not matter; I can contact either | 80 (55.2) |
|  | d) Any other: | 1 (0.7) |
| Which of the following practice(s) can make your animal(s) sick? | a) Clean animal sheds and tools | 6 (4.1) |
|  | b) Introduction of new animals in your animal herd/shed | 54 (36.5) |
|  | c) Absence of flies and tick in the shed/farm | 34 (23) |
|  | d) None of the above | 54 (36.5) |
| Which of the following do you think would be an important strategy in preventing and controlling infections in your animals? | a) Providing good quality feed and water | 7 (4.9) |
|  | b) Doing regular deworming and vaccination | 4 (2.8) |
|  | c) Regular advice from a veterinarian | 9 (6.3) |
|  | d) All of the above | 122 (85.9) |
| In what order should you take care of/handle/milk animals if sick animals are present? | a) Care for sick animal(s) before the healthy ones | 61 (43.3) |
|  | b) Care for healthy animal(s) before the sick ones | 59 (41.8) |
|  | c) a or b | 6 (4.3) |
|  | d) Order doesn't matter - you can go back and forth between healthy and sick animals | 15 (10.6) |
| How should you clean animal sheds or wash the utensils/buckets/cans used for milking animals or storing milk? | a) With water only | 17 (11.7) |
|  | b) With water and simple soap powder/solution | 83 (57.2) |
|  | c) With water and soap powder/solution containing disinfectant | 43 (29.7) |
|  | d) Removing dirt /refuse by sweeping only | 2 (1.4) |
| How long should you wait to allow an animal to sit down after milking: | a) 5 minutes | 6 (4.1) |
|  | b) 10 minutes | 57 (38.5) |
|  | c) 30 minutes | 60 (40.5) |
|  | d) The animal can sit immediately | 25 (16.9) |
| Which of the following statements is not correct: | a) There are several diseases which can spread from animals to humans | 20 (14.3) |
|  | b) There are several diseases which can spread from animals to humans and vice versa | 40 (28.6) |
|  | c) Animals cannot make you sick | 69 (49.3) |
|  | d) Diseases transmitted from animals to humans are known as Zoonotic diseases | 11 (7.9) |
| Which of the following is not a good practice and can make you sick: | a) Drinking raw milk of an animal | 21 (15.3) |
|  | b) Eating raw /undercooked meat of an animal | 4 (2.9) |
|  | c) Not washing hands after handling animals | 17 (12.4) |
|  | d) All of the above | 95 (69.3) |
| Which of the following is a correct statement: | a) Files and mosquitoes can transmit diseases only in humans | 8 (5.8) |
|  | b) There are some diseases which could be transmitted by inhalation in contaminated environment | 25 (18.2) |
|  | c) Ticks can transmit diseases only in animals | 25 (18.2) |
|  | d) All of the above | 79 (57.7) |
